# Structural Characteristics and Immunological Function of a New Non-Starch Polysaccharide from *Red Sprout* Taro

**DOI:** 10.3390/foods13223531

**Published:** 2024-11-05

**Authors:** Sha Luo, Yao Xiao, Asjad Ali, Qianglong Zhu, Nan Shan, Jingyu Sun, Shenglin Wang, Jianhui Xiao, Yingjin Huang, Qinghong Zhou

**Affiliations:** 1 Jiangxi Province Key Laboratory of Vegetable Cultivation and Utilization, College of Agronomy, Jiangxi Agricultural University, Nanchang 330045, China; rosalycake@163.com (S.L.); xiaoyao1990@jxau.edu.cn (Y.X.); longzhu2011@126.com (Q.Z.); shannan@jxau.edu.cn (N.S.); sunjingyu@jxau.edu.cn (J.S.); slinyx@126.com (S.W.); xiaojh666666@126.com (J.X.); 2 Queensland Department of Agriculture and Fisheries, P.O. Box 1054, Mareeba, QLD 4880, Australia; asjad.ali@daf.qld.gov.au; 3 Key Laboratory of Crop Physiology, Ecology, Genetic Breeding of the Ministry of Education, Nanchang 330045, China

**Keywords:** dietary fiber, inflammation, macrophages, phagocytic capacity, tuber crop

## Abstract

Taro is a tuber crop that is used for nutritional and medicinal purposes due to its abundance of non-starch polysaccharides (NSPs). *Red Sprout* taro is a local variety in Southern China, but the characteristics and bioactivities of its NSPs are currently unknown. In this study, NSPs were isolated from the corms of *Red Sprout* taro using hot-water extraction, ion-exchange chromatography, and ethanol precipitation; their molecular weight, monosaccharide composition, structural formulae, and immunomodulatory effects were examined. A novel NSP named *Colocasia esculenta* polysaccharide 1 (CEP1) was purified and characterized and was shown to mainly consist of glucose (60.49%) and galactose (25.92%) and have a molecular weight of 4556.272 kDa. The backbone of CEP1 consisted primarily of →4)-α-D-Glcp-(1→, →4,6)-β-D-Galp-(1→, and →3)-β-D-Galp-(1→ residues, with a branch consisting of the β-D-Glcp-(1→ residue. In addition, 25–400 µg/mL CEP1 was shown to have immunomodulatory effects on RAW264.7 macrophages. CEP1 not only increased cell viability, phagocytic capacity, inducible nitric oxide synthase secretion, and nitric oxide generation in RAW264.7 cells, but it also activated M1 and M2 macrophages to generate tumor necrosis factor α, interleukin 6, transforming growth factor β, and interleukin 10. These findings could lead to the use of CEP1 from Red Sprout taro as a possible immunomodulatory polysaccharide in functional foods.

## 1. Introduction

*Colocasia esculenta* (L.) Schott is one of the most important tuber crops in Africa and parts of Asia and is used as a staple food in some countries of these regions [1]. China has abundant genetic resources of taro and a long history of its cultivation [2]. The main cultivars of Chinese taro include Red Sprout taro, White Sprout taro, and Bun-long taro [3]. *C. esculenta* cv. Red Sprout taro is a local variety from Jiangxi Province, Southern China, that is highly nutritious, has a smooth and glutinous taste, and has excellent flesh quality [4]. Although taro corms contain low protein (1–2%) and fat (0.2%), taro provides good sources of carbohydrates (70–80% starch), fiber (0.8%), and ash (1.2%) [5]. In addition, taro is widely used as a nutritional and medicinal vegetable due to its abundance of non-starch polysaccharides (NSPs), mucilage, anthocyanins, vitamins, minerals, and niacin. These compounds have been proven to have antitumor, antimicrobial, antidiabetic, antihepatotoxic, and antimelanogenic effects [2,6,7].

Polysaccharides (also known as glycans) are composed of monosaccharides. They are fundamental components and physiologically active substances in living organisms [8]. Starch is the major polysaccharide in taro and has been extensively studied [3,9,10,11,12], yet only a few studies have been conducted on the purification of NSPs in *C. esculenta* [13,14,15,16,17]. Park et al., (2013) extracted and purified an NSP from edible Korean taro corm using cold-water extraction, ethanol precipitation, ion-exchange chromatography, and size-exclusion chromatography [16]. This NSP was composed of 64.4% neutral sugars and 35.6% uronic acid with a molecular weight of 200 kDa. Two NSPs (TSP-1 and TSP-2) were isolated and purified from the corms of Bun-long taro in China with a hot-water extraction method [15]. TPS-1 consisted of rhamnose (Rha, 8.5%), xylose (Xyl, 0.73%), glucose (Glc, 88.57%), and galactose (Gal, 2.56%), with a molecular weight of 10.502 kDa, while TPS-2 consisted of Rha (3.54%), arabinose (Arf, 1.6%), Glc (87.92%), and Gal (6.94%), with a molecular weight of 10.191 kDa [15]. In addition, NSPs were extracted from taro corms (pink cultivar from Fiji) in New Zealand using a new freeze–thaw method [13,14], comprising arabinose, galactose, glucose, and mannose. The functional groups of the NSPs included hydroxyl (–OH), carbonyl (C=O), carboxyl (-COOH), and methyl (–CH_3_) [13,14]. However, the characteristics of NSPs in Red Sprout taro, especially their structural formulae, have not been studied.

Polysaccharides have various bioactivities, such as immunomodulatory effects. Macrophages exhibit a crucial function in the immune system [18]. After activation, these cells can be polarized into two phenotypes, M1 and M2 macrophages, both of which are closely related to inflammatory responses [15,19,20]. M1 macrophages can induce inflammatory responses by producing tumor necrosis factor α (TNF-α), interleukin 6 (IL-6), and nitric oxide (NO). By contrast, M2-type macrophages, which are triggered by interleukin 4 (IL-4)/interleukin 13 (IL-13), can reduce inflammation [21]. Furthermore, polysaccharides from *C. esculenta*, *Alpiniae oxyphyllae*, *Cucumis metuliferus*, and *Passiflora foetida* can enhance the secretion of TNF-α and IL-6 and increase the production of inducible nitric oxide synthase (INOs) in macrophages [15,21,22,23]. However, the immunoregulation of taro NSPs is poorly understood. Therefore, it is worth studying the immunoregulation of macrophages by NSPs in Red Sprout taro.

In this study, a novel *C. esculenta* polysaccharide from Red Sprout taro was extracted, purified, and structurally characterized. Moreover, the immunoregulatory impact of this polysaccharide on RAW264.7 macrophages was studied with regard to cell viability, NO production, and cytokine secretion.

## 2. Materials and Methods

### 2.1. Extraction and Purification of Polysaccharides

Water extraction and alcohol precipitation were used to prepare crude polysaccharides. The corms of *Colocasia esculenta* cv. Red Sprout were acquired from Jiangxi Province. Briefly, the powders of the dried, peeled corms were mixed with distilled water (1:10, *w/v*) overnight and pass through a 100-mesh sieve. The residue was transferred to a new beaker. The filtrate was centrifuged to remove the starch. The supernatant was kept and mixed with the residue and heated to 70 °C for 3 h. The mixture was centrifuged to collect the supernatant. Four-fold volume of absolute alcohol was added to the supernatant to collect the crude polysaccharides. The crude polysaccharides were dissolved in ddH_2_O and deproteinized by 10 × 10^4^ U/g papain digestion overnight. After digestion, the solution of crude polysaccharides was defatted by the addition of petroleum ether to a concentration of 2% (*v*/*v*). The aqueous phase was collected, and a quarter volume of chloroform and butanol (chloroform: butanol = 4:1, *v*/*v*) was added to remove the residual proteins by mixing and centrifuging. The extracted polysaccharides were present in the aqueous phase and cleaned by the activated carbon and loaded into a diethylaminoethyl (DEAE)-cellulose Fast Flow column (1.6 × 40 cm, Buchi, Flawil, Switzerland) for ion-exchange chromatography. The polysaccharides were eluted with ddH_2_O and 0.1, 0.2, and 0.3 mol/L NaCl at 5.0 mL/min. Then, graded alcohol precipitation was used to collect the polysaccharides [24,25].

### 2.2. Characteristics of the Polysaccharides

#### 2.2.1. Determination of Physical Properties

After purification, the colour and the solubility were examined. The polysaccharide was dissolved at the concentrations of 1, 2, 3, 4, and 5% (*w*/*v*) and heated to a slight boil. The solutions were cooled to room temperature and kept at 4 °C for 24 h to determine whether the polysaccharide solutions formed a gel. In addition, the polysaccharide was dissolved at the concentrations of 2, 4, 8, and 10% (*w*/*v*), and the viscosity was examined by a rotary viscometer (Shanghai Changji geological instrument Co., Ltd., Shanghai, China) with the shear rate of 60 r/min at 20 °C.

#### 2.2.2. Molecular Weight (Mw) Determination

The polysaccharides were dissolved with 0.1 mol/L NaNO_3_ to a concentration of 1 mg/mL. Then, a 0.45 μm aperture was utilized to filter the samples. The filtrates were subjected to gel permeation chromatography-differential refractive Index detector- multi-angle laser light scatterer to determine the Mw distribution, number molar mass (Mn), and polydispersity (Mw/Mn) as described previously [23].

#### 2.2.3. Monosaccharide Composition Analysis

One millilitre of trifluoroacetic acid (2 mol/L) was added to 5 mg of polysaccharide and hydrolyzed at 121 °C for 2 h. To remove the extra trifluoroacetic acid, nitrogen gas and methanol were used to clean the samples three times, after which the samples were dissolved in ddHO_2_ and transferred to chromatographic bottles for further study. After that, the samples were subjected to anion-exchange chromatography as described previously [23]. Sigma-Aldrich Co. (St. Louis, MO, USA) provided the monosaccharide standards.

#### 2.2.4. Fourier Transform Infrared Spectroscopy (FT-IR) Analysis

Samples of 2 mg polysaccharide and 200 mg potassium bromide were compressed into tablets for FT-IR analysis by a Nicolet iZ-10 FT-IR spectrometer as described previously [21].

#### 2.2.5. Methylation Analysis

Polysaccharide (10 mg) was dissolved in 1 mL ddH_2_O, treated with 1 mL 50 mg/mL carbodiimide, and then mixed with 1 mL 2 mol/L imidazole. The mixtures were divided evenly into two portions. The samples were reacted with 12 mg/mL NaBH_4_ or NaBD_4_ for 3 h, after which glacial acetic acid was added. Immediately after dialysis and lyophilization, the ice-dried polysaccharides were dissolved in dimethyl sulfoxide and methylated with methyl iodide. The product was treated with 2 mol/L NH_4_OH and 0.5 mol/L NaBD_4_ for 2.5 h. The reduced product was dried using nitrogen and acetylated with acetic anhydride. The glycosidic bonds of the polysaccharides were analyzed with a gas chromatograph–mass spectrometer (Agilent 7890A-5977B, Agilent Technologies, Santa Clara, CA, USA) as described previously [23].

#### 2.2.6. Nuclear Magnetic Resonance (NMR) Spectroscopy

For NMR spectroscopy, the samples were thoroughly dissolved in D_2_O and mixed with a 40 mg/molar concentration of polysaccharide. The solution was then loaded on an NMR spectrometer to scan heteronuclear single quantum correlation (HSQC), 13C-NMR, correlation spectroscopy (COSY), 1H-NMR, nuclear Overhauser effect spectroscopy (NOESY), and heteronuclear multiple-bond correlation (HMBC) spectral images using a Bruker AVANCE HD III 600 MHz spectrometer (Germany) with the liquid probe QXI1H/31P/13C/15N5 mm quadratically resonating at 25 °C.

### 2.3. Immunomodulatory Activity of CEP1

#### 2.3.1. Cell Culture and Reagents

The culture medium for RAW264.7 cells was prepared using 90% Dulbecco’s modified Eagle’s medium (DMEM; Thermo Fisher Scientific, Inc., Wilmington, DE, USA) supplemented with 10% heat-inactivated fetal bovine serum (Thermo Fisher Scientific, Inc.), 1% penicillin (100 U/mL; Thermo Fisher Scientific, Inc.), and streptomycin (100 µg/mL; Thermo Fisher Scientific, Inc.). The cultivation parameters for the cells were 37 °C in a CO_2_ incubator containing 5% CO_2_.

#### 2.3.2. Cell Viability Assay

The RAW264.7 macrophages (Cellosaurus accession number: CVCL_0493) were cultured for 6 h and treated with 5 μg/mL lipopolysaccharides (LPS, positive control), DMEM (control), and 25, 50, 100, 200, 400, and 800 μg/mL CEP1 for 24 h in a CO_2_ incubator at 37 °C and 5% CO_2_. The viability of the RAW264.7 macrophages was assessed using a methyl thiazole tetrazolium (MTT) assay kit (Beyotime, Shanghai, China) as per the user manual.

#### 2.3.3. Cell Phagocytic Activity Assay

The phagocytic activity was assessed by using a Vybrant™ Phagocytosis test kit (Thermo Fisher Scientific). Briefly, the RAW264.7 macrophages were cultured for 6 h and were treated with 1 μg/mL LPS (positive control), DMEM (control), and 25, 100, and 400 μg/mL CEP1 for 24 h in a CO_2_ incubator at 37 °C and 5% CO_2_. Then, the cells were treated with *Escherichia coli* labelled with fluorescein 5-isothiocyanate for 2 h. The fluorescence of all the groups was measured by a fluorescence plate reader using ~480 nm excitation, ~520 nm emission. The phagocytic activity was calculated according to the user manual and normalized to the control group.

#### 2.3.4. Effects of CEP1 on M1 Macrophages

The RAW264.7 macrophages underwent culture for 6 h and were treated with 1 μg/mL LPS (positive control), DMEM (control), and 25, 100, and 400 μg/mL CEP1 for 24 h in a CO_2_ incubator at 37 °C and 5% CO_2_. The protein levels of IL-6 and TNF-α were measured by a mouse enzyme-linked immunosorbnent assay (ELISA) kit (Beyotime Biotechnology, Shanghai, China). The NO concentration was measured by an NO assay kit (Beyotime Biotechnology). The protein level of INOS was quantified using a mouse INOS ELISA Kit (Abcam Limited, Cambridge, UK) according to the user manual.

#### 2.3.5. Effects of CEP1 on M2 Macrophages

The RAW264.7 macrophages were cultured for 6 h and treated with 20 ng/mL IL-4 (positive control), DMEM (control), and 25, 100, and 400 μg/mL CEP1 for 24 h in a CO_2_ incubator at 37 °C and 5% CO_2_. A mouse interleukin 10 (IL-10) ELISA kit (Beyotime Biotechnology) was used to detect the protein level of IL-10. The protein level of transforming growth factor β (TGF-β) was quantified with a mouse TGF-β ELISA kit (Beyotime Biotechnology) according to the user manual.

## 3. Results and Discussion

### 3.1. CEP1 Characterization

#### 3.1.1. Extraction, Purification, and Fractionation of Polysaccharides

**Figure 1:**
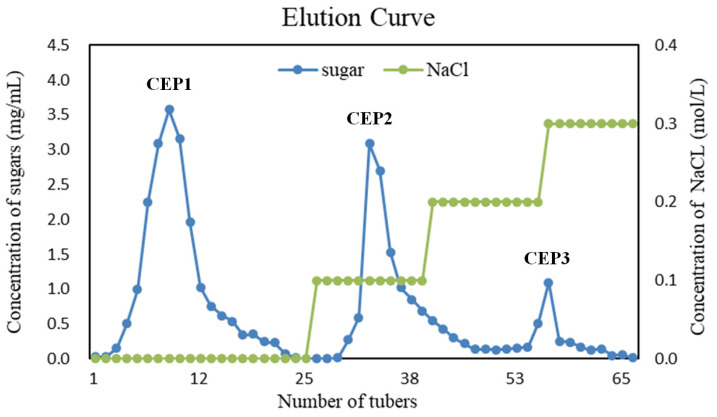
The elution profile of the crude polysaccharides of *C. esculenta* cv. Red Sprout on a DEAE-52 cellulose column. CEP1, CEP2, and CEP3 were collected from tube 3 to tube 19, from tube 32 to tube 42, and from tube 55 to tube 56, respectively.

In total, 400 g powders of the dried, peeled corms were used for the isolation of the crude polysaccharides, and 21.2 g crude polysaccharides were obtained. The yield of crude polysaccharides was 5.30% (*w*/*w*), which is higher than the yield of 4.12% (*w*/*w*) reported previously [16]. Subsequently, 1.8 g purified polysaccharides were obtained, which contain three polysaccharides, CEP1 (0.6 g), CEP2 (1.1 g), and CEP3 (0.1 g), eluted with ddH_2_O, 0.1 mol/L NaCl, and 0.2 mol/L NaCl, respectively, with polysaccharide purities of 48.1%, 13.4%, and 15.4%, respectively (Figure 1). Since CEP1 had the highest polysaccharide purity, this study focused mainly on this polysaccharide. CEP1 is white in color and has a solubility of 12% in hot water. The viscosity of CEP1 solution increases with the mass fraction. The viscosity of the 2, 4, 8, and 10% CEP1 solutions are 16, 22, 47, 105, and 170 mPa·s, respectively. However, CEP1 could not form gel even when the concentration reached 5.0%, indicating that the polysaccharide could not form gel by itself.

Mw and Mn are two important parameters used to evaluate the physicochemical properties of polysaccharides. The Mw and Mn of the polysaccharide CEP1 were 4556.272 kDa and 3034.895 kDa, respectively. However, a previous study reported that the Mw of the taro polysaccharides was 10.191 kDa and 200 kDa [15,16]. The differences in the Mw of these NSPs might be due to unique growing areas, species, extraction methods, and/or purification methods. Previously, polysaccharides with a high Mw of 1260–2330 kDa were purified from edible bulbs [24]. Since Mw is closely associated with the solubility of polysaccharides, those with high Mw are difficult to dissolve [26]. In this study, we also found very viscous crude polysaccharides, which suggests that the polysaccharides in the taro had a high Mw.

#### 3.1.2. Monosaccharide Composition

CEP1 contains glucose, galactose, mannose, arabinose, xylose, and fucose with molar proportions of 60.49%, 25.92%, 5.13%, 4.04%, 0.64%, and 0.28%, respectively (Table 1). The monosaccharide composition of CEP1 found in this study was different from that of polysaccharides identified in Bun-long taro cultivated in Guangdong, China reported by Li et al., (2018) [15]. These discrepancies might be linked to differences in the type of taro, planting location, extraction methods, and/or purification methods used [17].

#### 3.1.3. FT-IR Analysis

**Figure 2:**
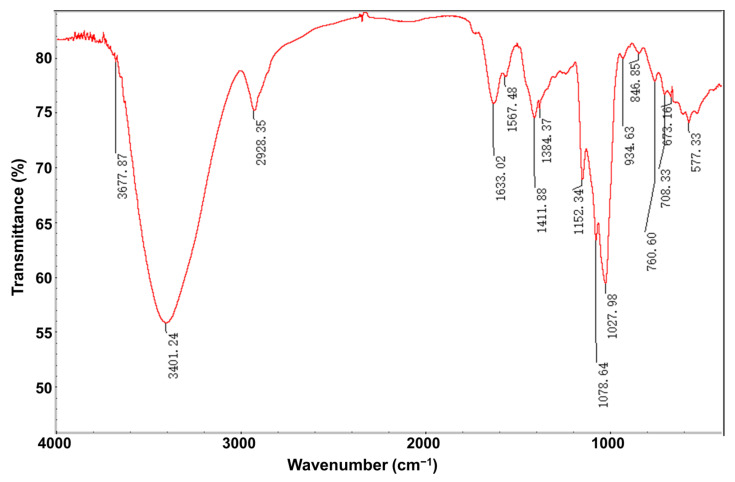
The FT-IR spectra of the polysaccharide CEP1.

The FT-IR results showed that the broadband at 3401.24 cm^−1^ for CEP1 was attributed to the stretching vibration of O-H (Figure 2), which indicated the presence of carbohydrates [27]. However, the weak absorption band at approximately 2928.35 cm^−1^ was probably caused by the C-H stretching vibration in the sugar ring at their original length (Figure 2). Furthermore, the absorption peak shown at 1633.02 cm^−1^ denoted uronic acids (Figure 2), which was attributed to the stretching vibration of C=O [28]. The absorption bands from 1000.0 to 1200.0 cm^−1^ represented the structural arrangements of C-O-H and C-O-C (Figure 2). Additionally, the absorption peak at 1078.64 cm^−1^ indicated the pyranose ring of the glucosyl residue in this sugar (Figure 2). Furthermore, α-glycosidic bonds and β-glycosidic bonds were observed in the absorbance peaks at 934.63 cm^−1^ and 846.85 cm^−1^, respectively (Figure 2) [29]. The FT-IR findings demonstrated that CEP1 has a typical sugar group.

#### 3.1.4. Results of Methylation Analysis

The result of our methylation analysis showed that CEP1 has ten linkages—t-Ara(f), t-Glc(p), t-Gal(p), 3-Gal(p), 6-Man(p), 6-Glc(p), 4-Glc(p), 3,4-Glc(p), 4,6-Glc(p), and 2,3,6-Man(p)—in all the sugar residues, with molar percentages of 0.776%, 17.635%, 5.856%, 8.123%, 2.328%, 2.989%, 48.974%, 0.542%, 9.512%, and 3.263%, respectively (Table 2). Among these linkages, 4-Glc (p) accounted for the largest percentage of the total Glc linkages, indicating its major role in building the backbone of CEP1. Moreover, these results further confirmed the monosaccharide composition of CEP1 (Table 1).

#### 3.1.5. NMR Analysis

**Figure 3:**
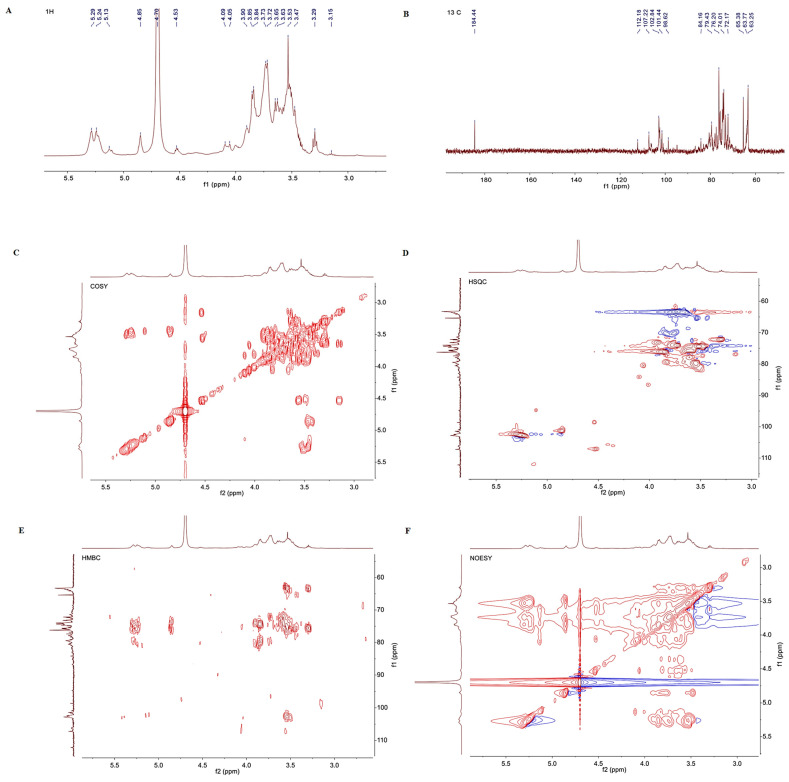
Nuclear magnetic resonance (NMR) spectral analysis of CEP1 from C. esculenta cv. Red Sprout. (**A**) 1H-NMR of CEP1; (**B**) 13C-NMR of CEP1; (**C**) 1H-1H correlation spectroscopy (COSY) of CEP1; (**D**) heteronuclear single quantum correlation (HSQC) of CEP1; (**E**) heteronuclear multiple-bond correlation (HMBC) of CEP1; (**F**) nuclear Overhauser effect spectroscopy (NOESY) of CEP1.

NMR analysis was utilized to further interpret the structure of CEP1, as 1D-NMR (1H- and 13C-) can be used to confirm the configuring glycosidic bonds in polysaccharides [30]. The hydrogen spectral signal of CEP1 was concentrated at 3.1 and 5.5 ppm. A total of six anomeric proton chemical shifts at 5.29, 4.86, 4.53, 4.54, 5.24, and 5.12 ppm were assigned to H-1 (Figure 3A). The anomeric signals in the 5.1–5.8 ppm region represent α-heteromeric hydrogen, and those in the 4.4–5.0 ppm region represent β-heteromeric hydrogen [22]. Therefore, the chemical shifts at 5.29 ppm (residue A), 5.24 ppm, and 5.12 ppm (residue E) in the CEP1 polysaccharide were attributed to α-heteromeric hydrogen, while the signals at 4.86 ppm (residue B), 4.53 ppm (residue C), and 4.54 ppm (residue D) were attributed to β-heteromeric hydrogen (Figure 3A and Table 3). The heterogeneous hydrogen signals were concentrated in the ppm region ranging from 3.1 to 4.2 ppm. The chemical shifts of H2–H6 were assigned to sugar residues A, B, and C utilizing both COSY and HSQC spectra.

Primarily, the heteromeric carbon signals of the polysaccharides in the 13C NMR spectrum were concentrated at 92–115 ppm, and the anomeric carbon signals at 112.18, 107.22, 102.84, 101.44, 98.62, and 94.87 ppm indicated the presence of C-1 residues in CEP1 (Figure 3B). In addition, a signal peak was observed at 184.44 ppm, which is usually attributed to the free carboxyl group. It was inferred from the monosaccharide composition results that this signal was the C=O carbon in glucuronic acid. However, due to its low content, the chemical shifts could not be attributed in detail.

The COSY spectrum is mainly used to analyze adjacent or interphase proton hydrogen-related signals [30]. The H1/H2, H2/H3, H3/H4, H4/H5, and H5/H6 of →4)-α-D-Glcp-(1→ were identified by the cross-peaks at 5.29/3.52, 3.52/3.86, 3.86/3.55, 3.55/3.73, and 3.73/3.74 ppm, respectively. The cross-peaks at 4.53/3.37, 3.37/3.92, 3.92/3.84, 3.84/3.72, and 3.72/3.29 ppm indicated the H1/H2, H2/H3, H3/H4, H4/H5, and H5/H6 of → 4,6)-β-D-Galp (1→, correspondingly. Furthermore, signals corresponding to →3)-β-D-Galp-(1→ (from H1 to H6) were detected at 4.54, 3.15, 4.06, 3.64, 3.77, and 3.54 ppm (Figure 3C and Table 3).

The HSQC spectrum is used to detect remote coupling correlations between C-H with high sensitivity [30]. The HSQC spectrum showed the presence of five cross-peaks at 5.29/102.42 (residue A), 4.86/101.45 (residue B), 4.53/107.24 (residue C), 4.54/98.61 (residue D), and 5.12/94.87 ppm (residue E) (Figure 3D and Table 3). The residual A, B, C, D, and E were ascertained to be →4-α-D-Glcp-(1→, β-D-Glcp-(1→, → 4,6)-β-D-Galp-(1→, →3)-β-D-Galp-(1→, and α-D-Galp-(1→ according to the bonding structure (methylation) information and heterotopic signals (Figure 3D and Table 3).

The result of HMBC validated the linkage of glycoside residues in CEP1. H1 (5.29 ppm, residue A) had cross-peaks with C4 (79.81 ppm, residue A) and C4 (79.42 ppm, residue C). There were also cross-peaks between C1 (102.42 ppm, residue A)/H4 (3.55 ppm, residue A), C1 (101.45 ppm, residue B)/H6 (3.54 ppm, residue C), H1 (4.53 ppm, residue C)/C3 (80.57 ppm, residue D), and C1 (107.24 ppm, residue C)/H3 (4.06 ppm, residue D) (Figure 3E). Moreover, the order of connection for each residue in the polysaccharide was determined based on the NOESY spectrum. There were cross-peaks between the H1 of residue A and the H4 of residues A and C. In addition, cross-peaks between H1 (residue C)/H3 (residue D) and H1 (residue D)/H4 (residue A) were visualized (Figure 3F).

**Figure 4:**
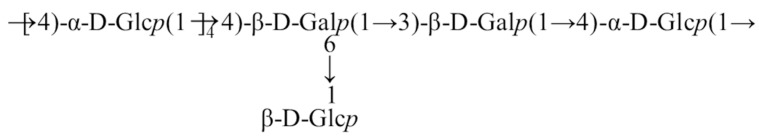
Projected structure of CEP1.

The polysaccharide was determined to comprise → 4)-α-D-Glcp-(1→, →4,6)-β-D-Galp-(1→, and → 3)-β-D-Galp-(1→ as the main chain, with the end sugar β-D-Glcp-(1→ linked to position 6 of residue C to form a branched chain through the use of 1D-NMR, 2D-NMR, and the reported data (Table 3). No relevant connections were identified at the α-D-Galp-(1→) position at low concentrations. Therefore, the speculated structural formula of CEP1 is displayed in Figure 4. Previous studies have only identified the chemical shifts of polysaccharides in taro [15]. In this study, novel NSPs in Red Sprout taro were identified, and their structural formulae were first proposed.

### 3.2. RAW264.7 Macrophage Viability Was Enhanced by CEP1

**Figure 5:**
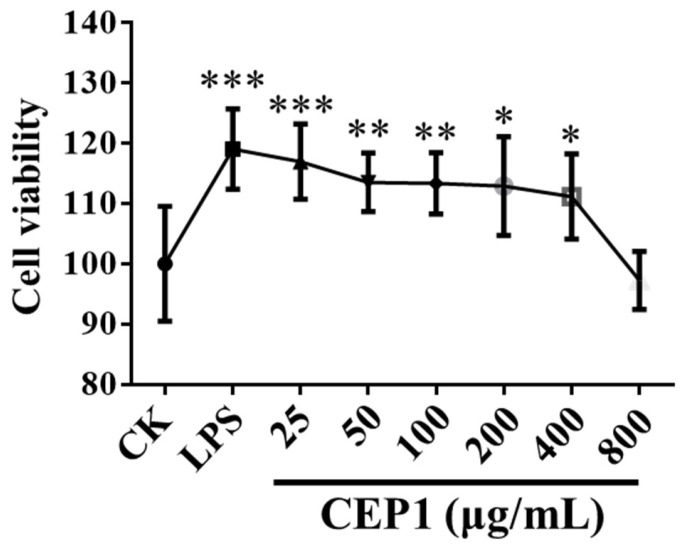
Impacts of CEP1 on the viability of RAW264.7 macrophages. RAW264.7 macrophages were cultured for 6 h and treated with 5 μg/mL LPS (positive control), DMEM (control), and 25, 50, 100, 200, 400, and 800 μg/mL CEP1 for 24 h at 37 °C in 5% CO_2_. Cell viability was examined using the methyl thiazole tetrazolium (MTT) method. The experiments were repeated six times. The data are expressed as the mean ± standard deviation. * *p* < 0.05, ** *p* < 0.01, and *** *p* < 0.001 by one-way ANOVA and Dunnett’s test.

The immunomodulatory effect is an important bioactive property of polysaccharides [31]. These immunomodulatory effects have previously been evaluated using macrophages as an ideal cell model [17]. Previous studies have shown that 250–500 μg/mL polysaccharides from *Smilax glabra Roxb*, *Grateloupia livida*, and *Cyclocarya paliurus* increased the viability of RAW264.7 macrophages [21,32,33]. The polysaccharides from Bun-long taro failed to exhibit an impact on the viability of RAW264.7 macrophages [15]. Here, we found that 25 to 400 μg/mL CEP1 increased the viability of the above macrophages, while 800 μg/mL CEP1 did not have an effect (Figure 5). These results suggest that CEP1 is more effective at promoting the immunomodulation of macrophages than the previously identified polysaccharides from *Smilax glabra Roxb*, *G*. *livida*, and *C*. *paliurus*.

### 3.3. CEP1 Enhanced the Phagocytic Capability of RAW264.7 Macrophages

**Figure 6:**
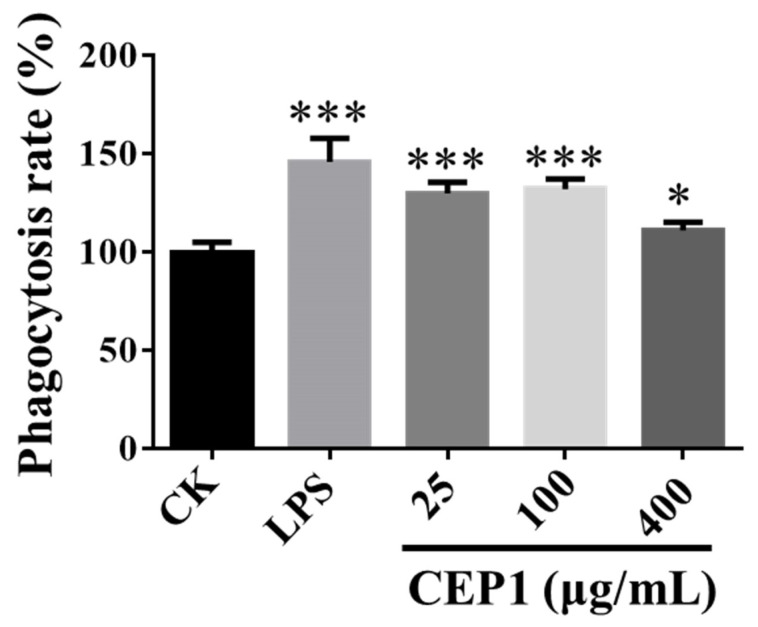
Impacts of CEP1 on the phagocytic activity of RAW264.7 macrophages. RAW264.7 macrophages were cultured for 6 h and treated with 1 μg/mL LPS (positive control), DMEM (control), and 25, 100, and 400 μg/mL CEP1 for 24 h at 37 °C and 5% CO_2_. The phagocytic activity of the RAW264.7 macrophages was assessed with a Vybrant™ Phagocytosis test kit. The experiments were performed six times. The data are expressed as the mean ± standard deviation. * *p* < 0.05 and *** *p* < 0.001 by one-way ANOVA and Dunnett’s test.

The phagocytic capacity of macrophages is essential for defending cells against pathogen infections [34]. Compared to the control group, CEP1 at concentrations of 25, 100, or 400 μg/mL remarkably increased the phagocytic activity of RAW264.7 macrophages (Figure 6). RAW264.7 macrophages were found to enhance phagocytic activity following CEP1 intervention. In a previous study, the enhancing effect of 500 and 1000 μg/mL Bun-long taro polysaccharides on the phagocytic activity of RAW264.7 macrophages was identified [15]. Similarly, we detected an enhancing effect of 25 μg/mL CEP1 on the phagocytic activity of the aforementioned macrophages. These results suggest that CEP1 is more effective at enhancing the phagocytic activity of RAW264.7 cells than Bun-long taro polysaccharides.

### 3.4. CEP1 Increased the INOS, NO, IL-6, and TNF-α Produced by M1 Macrophages

**Figure 7:**
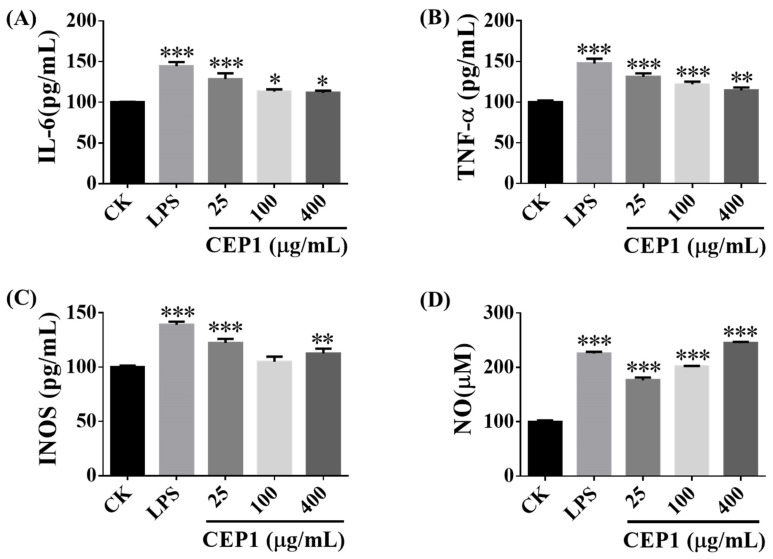
Activation impacts of CEP1 on M1 macrophages. Impact of CEP1 on the phagocytic activity of RAW264.7 macrophages. RAW264.7 macrophages were cultured for 6 h and treated with 1 μg/mL LPS (positive control), DMEM (control), and 25, 100, and 400 μg/mL CEP1 for 24 h at 37 °C and 5% CO_2_. The protein levels of IL-6 (**A**), TNF-α (**B**), and INOS (**C**) as well as NO production (**D**) were analyzed via ELISA kits. The experiments were repeated three times. The data are expressed as the mean ± standard deviation. * *p* < 0.05, ** *p* < 0.01, and *** *p* < 0.001 by one-way ANOVA and Dunnett’s test.

M1 macrophages participate mainly in pro-inflammatory responses; they are crucial for the elimination of pathogens, viral infections, and cancerous tissue [35]. Polysaccharides can induce M1 macrophages to produce NO and secrete IL-6, TNF-α, and INOS [17]. Li et al., (2018) showed that 125 μg/mL polysaccharides from Bun-long taro could activate macrophages to secrete NO, TNF-α, and IL-6 [15]. The present study demonstrated that 25–400 μg/mL CEP1 enhanced the secretion of IL-6 (Figure 7A), TNF-α (Figure 7B), and INOS (Figure 7C) and the generation of NO (Figure 7D) in RAW264.7 macrophages. However, 100 μg/mL CEP1 did not affect the secretion of INOS (Figure 7C). These results suggest that CEP1 is more effective at activating M1 macrophages than Bun-long taro polysaccharides.

### 3.5. CEP1 Increased the TGF-β and IL-10 Secreted by M2 Macrophages

**Figure 8:**
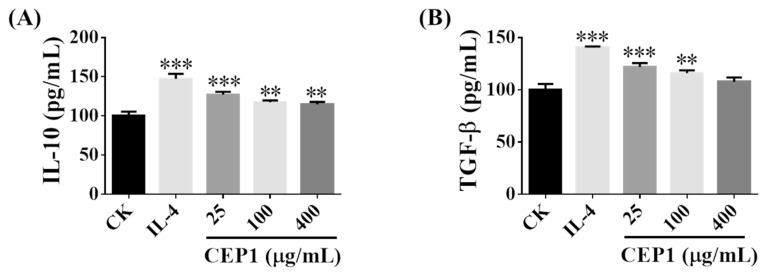
Activating impacts of CEP1 on M2 macrophages RAW264.7 macrophages were cultured for 6 h and treated with 20 ng/mL IL-4 (positive control), DMEM (control), and 25, 100, and 400 μg/mL CEP1 for 24 h at 37 °C and 5% CO_2_. The protein levels of IL-10 (**A**) and TGF-β (**B**) were quantified using ELISA kits. The experiments were triplicated. The data are expressed as the mean ± standard deviation. ** *p* < 0.01, and *** *p* < 0.001 by one-way ANOVA and Dunnett’s test.

M2 macrophages participate mainly in anti-inflammatory responses and are also induced by IL-4 and IL-13. M2-type microglobulin cells secrete IL-10 and TGF-β, which can reshape and transport tissues, thereby promoting the secretion of anti-inflammatory agents [36,37]. In this study, we found that 25–400 μg/mL CEP1 substantially enhanced the generation of IL-10 (Figure 8A). In addition, 25 and 100 μg/mL CEP1 increased the secretion of TGF-β, whereas 400 μg/mL CEP1 had no effect (Figure 8B). These results suggest that CEP1 can activate a Th2-type immune response by activating macrophages at proper doses.

## 4. Conclusions

This study identified a novel polysaccharide (CEP1) in Red Sprout taro. CEP1 primarily comprises glucose (60.49%) and galactose (25.92%) and has a molecular weight of 4556.272 kDa. The backbone of CEP1 predominantly comprised → 4)-α-D-Glcp-(1→, →4,6)-β-D-Galp-(1→, and →3)-β-D-Galp-(1→ residues, with a branch consisting of the β-D-Glcp-(1→ residue. In addition, CEP1 increased the viability as well as the phagocytic capacity of RAW264.7 cells. It activated M1 and M2 macrophages by enhancing the secretion of cytokines and increasing the protein level of INOS and NO production. These findings could lead to the use of CEP1 from C. esculenta cv. Red Sprout taro as a possible immunomodulatory polysaccharide in functional foods.

## Figures and Tables

**Table 1:** Monosaccharide compositions (%) of CEP1.

Monosaccharide Compositions	Ratio (%)
Glucose	60.49
Galactose	25.92
Mannose	5.13
Arabinose	4.04
Xylose	0.64
Fucose	0.28

**Table 2:** The glycosidic linkage pattern of CEP1 based on methylation analysis.

Retention Time (min)	Type of Linkage	Methylated Sugars	Molar Ratios (%)
5.71	t-Ara(f)	1,4-di-O-acetyl-2,3,5-tri-O-methyl arabinitol	0.776
8.589	t-Glc(p)	1,5-di-O-acetyl-2,3,4,6-tetra-O-methyl glucitol	17.635
9.568	t-Gal(p)	1,5-di-O-acetyl-2,3,4,6-tetra-O-methyl galactitol	5.856
12.446	3-Gal(p)	1,3,5-tri-O-acetyl-2,4,6-tri-O-methyl galactitol	8.123
13.168	6-Man(p)	1,5,6-tri-O-acetyl-2,3,4-tri-O-methyl mannitol	2.328
13.322	6-Glc(p)	1,5,6-tri-O-acetyl-2,3,4-tri-O-methyl glucitol	2.989
13.674	4-Glc(p)	1,4,5-tri-O-acetyl-2,3,6-tri-O-methyl glucitol	48.974
15.831	3,4-Glc(p)	1,3,4,5-tetra-O-acetyl-2,6-di-O-methyl glucitol	0.542
17.889	4,6-Glc(p)	1,4,5,6-tetra-O-acetyl-2,3-di-O-methyl glucitol	9.512
20.916	2,3,6-Man(p)	1,2,3,5,6-penta-O-acetyl-4-O-methyl mannitol	3.263

**Table 3:** Chemical shift assignment of glycosidic linkages in CEP1.

Code	Glycosyl Residues	Chemical Shifts (ppm)					
		H1/C1	H2/C2	H3/C3	H4/C4	H5/C5	H6a,b/C6
A	→4)-α-D-Glcp-(1→	5.29	3.52	3.86	3.55	3.73	3.74,3.66
		102.42	74.54	76.15	79.81	74.05	63.25
B	β-D-Glcp-(1→	4.86	3.47	3.92	3.3	3.74	3.75
		101.45	74.49	73.16	72.22	74.05	63.92
C	→4,6)-β-D-Galp-(1→	4.53	3.57	3.92	3.84	3.72	3.29,3.54
		107.24	74.71	73.16	79.42	74.14	72.26
D	→3)-β-D-Galp-(1→	4.54	3.15	4.06	3.64	3.77	3.54
		98.61	76.98	80.57	75.61	73.98	65.48
E	α-D-Galp-(1→	5.12	3.44	n.d	3.58	n.d	3.44
		94.87	n.d	n.d	75.77	n.d	65.33

## Data Availability

The original contributions presented in the study are included in the article, further inquiries can be directed to the corresponding authors.
